# Assessment of *Chlorella vulgaris* as a biological control agent against tortoise tick *Hyalomma aegyptium* (Acari: Ixodidae) in Egypt

**DOI:** 10.1038/s41598-025-13971-8

**Published:** 2025-08-15

**Authors:** Mohammed Okely, Asmaa Ali Baioumy Ali

**Affiliations:** 1 Entomology Department, Faculty of Science, Ain Shams University, Abbassia, Cairo, 11566 Egypt; 2 Zoology Department, Faculty of Science, Ain Shams University, Abbassia, Cairo, 11566 Egypt

**Keywords:** Algae, Biocontrol, Egypt, Ixodids, Ticks, Biological techniques, Cell biology, Structural biology, Zoology, Pathogenesis

## Abstract

*Hyalomma aegyptium* is a three-host tick species parasitizing mainly tortoises in Asia, North Africa, and the Middle East. It serves as a carrier for various pathogenic bacteria and protozoa that pose threats to humans, wildlife, and domestic animals. Ticks control using chemical acaricides has negative effects to the environment and animal and human health, residues in animal products and leading to resistant ticks. So safe, eco-friendly, and cost-effective methods must be alternatively used. The green microalga *Chlorella vulgaris* is rich in proteins, lipids, carbohydrates and vitamins. It is used in biofuel production, wastewater treatment, and as a biofertilizer. It is used in pharmaceutical drugs with many beneficial characteristics. Examination of collected specimens in the present study ensured that they were identified as *H. aegyptium* nymphs. Using the powdering method, nymphs were treated with *Chlorella* and observed for 18 days. The results showed that the effect began 4 days after treatment, the mortality percentage reached 80%, and delayed molting period with only 20% molted into males. Morphological observations using light and scanning electron microscopes revealed a stiffened nymph body after treatment with a highly damaged capitulum, integument, and legs. Integument semithin sections showed thin, disorganized cuticle with damaged layers and destructed epidermal cells after treatment. No signs of new cuticle formation were noticed. The effect of *Chlorella* was either mechanical through powder particles or physiological through its effect on organs. This study may provide valuable information to help in the development of new methods to control ticks and/or improve the existing ones, allowing the creation of methods which do not induce resistance in ticks, and that are less toxic to the environment and non-target organisms.

## Introduction

Ticks are hematophagous worldwide distributed ectoparasites of economic consequences^1–3^. They enhance the impact on the host through direct damage or indirect virtue of being carriers of infectious protozoan, rickettsial, and viral diseases^4–8^. They also transmit several human diseases such as Lyme disease, Crimean-Congo hemorrhagic fever, Kayasanur Forest disease, viral borne encephalitis, and zoonotic illnesses^9–11^. *Hyalomma*, *Rhipicephalus*, and *Amblyomma* are tropical areas’ most economically important ixodid tick genera^12^.

*Hyalomma aegyptium* (Linnaeus, 1758) is the dominating tick in Palearctic tortoises of *Testudo genus*; however, adults are also infrequently obtained from hares and hedgehogs^13^. Immature stages are considered a less specific host than adults collected from various animals such as tortoises, lizards, birds, small mammals, and humans^14,15^. This species is a three-host tick with a specific distribution in North Africa, Balkan countries, the Middle East, Central Asia, Iran, Afghanistan, and Pakistan^16–19^. Although several pathogens have been detected in *H. aegyptium*^20–23^*Hemolivia mauritanica*, *Hepatozoon kisrae* and *Coxiella burnetiid* were proven to be transmitted by this tick species^24,25^.

Ticks control using synthetic acaricides poses a risk to the environment and human health *and* may lead to residues in animal products^26^. Furthermore, ticks are becoming resistant to many acaricides^27,28^. In order to effectively manage ticks, compounds must have distinct mechanisms of action and be safe for the environment and host without producing resistant strains^11,29,30^. Numerous studies have demonstrated the advantages of employing natural plant extract to control ticks instead of synthetic acaricides^31–33^.

*Chlorella vulgaris* is a spherical green microalga with rapid growth rate^34,35^. The main components of its biomass are proteins, lipids, and carbohydrates, with the presence of pigments, vitamins, and other minor components^36^. This alga can thrive in wastewater as it can resilience in adverse conditions^35,37^. It is used in biofuel production^38–40^ wastewater treatment^39,40^ lowering greenhouse gas emissions^41^ and cosmetic industry^42^ additionally it acts as biofertilizer^43^. Its biocidal effects has been studied earlier.

Although microalgae-based technology offers a challenging, the previous properties, in addition to eco-friendly and cost-effective ones, allow them to be a sustainable option for energy production and environmental remediation^40^. Therefore, the purpose of this work is to investigate the effects of the microalga *C. vulgaris* on the development of *H. aegyptium* nymphs (either biologically or histologically) found on tortoises as a biological control agent, for the first time in Egypt.

## Materials and methods

### Tick origin and collection

Ticks (*n* = 225) were collected from the African spurred tortoise *Centrochelys sulcate* (endangered species comes from Africa) (*n* = 50) and reared in a farm located in Giza (GPS location: 30.0487 N, 31.0959 E). They were transferred to Medical Entomology Laboratory, Entomology Department, Faculty of Science, Ain Shams University and reared inside an incubator at 28 ± 2 ˚C and 75 ± 5% RH until the beginning of the experiment.

### Tick identification and imaging

Specimens were examined using CZM4Stereo Microscope (Labomed, Fremont, CA, USA) and identified according to taxonomic keys and diagnostic features^44,45^. Then they photographed using MU1000 10MP microscopic camera (AmScope, Irvine, CA, USA) fixed on the microscope in Medical Entomology Laboratory, Entomology Department, Faculty of Science, Ain Shams University within 24 h from collection. In addition, some were allowed to molt into adult stages to ensure accurate identification, as identification keys for the immature stages of this species are limited^45^.

### Algae collection, growth, and powder Preparation

*Chlorella vulgaris* Beyerinck purified strains were acquired from the Algal Biotechnology Unit, Fertilization Technology Department, National Research Centre, Cairo, Egypt. Then they transferred to Algae Unit, Botany Department, Faculty of Science, Ain Shams University, Cairo, Egypt for growth and preparation.

To get the right inoculum, the chosen isolate was cultivated in BG-II nutrient solution under ideal circumstances^46^. Daylight bulbs (5 × 40w) with reflexes from one side gave constant lighting with an intensity of around 120µ.e. Through a 3 mm polyethylene tube that was terminated by a compact sand distributor, aeration was accomplished using compressed air and free oil from the top hold. Throughout the whole incubation time, room temperature (27 ± 2 °C) was measured. 2 L of the algal broth was incubated in completely transparent polyethylene bags that were 75 cm long, 5 cm wide, and 100 µ thick^47^. After the growth peaked, the biomass was removed using a cooling centrifuge (RUNNE HEIDBERG model RSV-20) and cleaned twice to get rid of any remaining nutrients. Then biomass was oven-dried at 60 ˚C overnight.

### Tick treatment

The collected fed tick nymphs were used in the treatment method. Three trials were done for each untreated and treated group, with ten tick specimens each. Treated ticks were powdered by the alga (0.1 g/tick - minimum amount estimated to cover the surface of the tick’s body) at Invertebrate Laboratory, Zoology Department, Faculty of Science, Ain Shams University, Cairo, Egypt. Untreated groups (control groups) were left unpowdered. Both groups were reared in glass vials and kept inside the incubator at 28 ± 2 °C and 75 ± 5% RH. Treated ticks were compared with untreated ones and were examined for 18 days (the period after which no changes occurred).

### Biological studies

To study the biological parameters, this section was done in Invertebrate Laboratory, Zoology Department, Faculty of Science, Ain Shams University, Cairo, Egypt. Tick specimens were examined daily for mobility and mortality. The percentage of ticks that were mobile during the study period was used to calculate mobility (number of mobile nymphs/total number x 100). Soft forceps were used to press the specimens multiple times in order to check their movement. Tick specimens were considered dead if they didn’t move their legs.

Percentage of molting (number of molted nymphs/total number x 100) and duration of molting (days from fed to molt) were calculated.

The efficacy of alga (E) was assessed in ticks 18 days after treatment following the equation E = [B-T/ B] 100, where B is the mean number of surviving ticks in the control group and T is the mean number of surviving ticks in the treated group^48^.

### Statistical analysis

For evaluation of biological parameters, 30 tick specimens were considered untreated (control), and other 30 were treated with alga powder. Means and standard errors were calculated using Microsoft Excel 2010 software. For the relationships between control and treated specimens, one-way ANOVA test was used followed by Schaff’s test^49^. The effect of the alga on ticks was evaluated when significant differences were considered (*p* < 0.05).

### Histological studies

After 11 days after feeding and/or treatment, live nymphs were examined using a scanning electron microscope and light microscope. Treated specimens were compared with untreated ones (*n* = 5 in each examination).

#### Morphological studies

##### Light microscopy (Whole specimens)

For general observation and comparison, both untreated and treated nymphs were examined and photographed using MU1000 10MP microscopic camera (AmScope, Irvine, CA, USA) fixed on CZM4Stereo Microscope (Labomed, Fremont, CA, USA) in Medical Entomology Laboratory, Entomology Department, Faculty of Science, Ain Shams University.

##### Scanning electron microscopy (SEM) (Whole specimens)

Nymphs were fixed for 2 h in 3% cold fresh glutaraldehyde. They were washed for 30 min. in phosphate buffer, then dehydrated in ascending series of ethanol. Specimens were directly subjected to critical point drying, attached with double-sided carbon tap to aluminum stubs, coated with gold in a sputter-coating apparatus, and then examined under a JEOL scanning electron microscope at Desert Research Center, Matareya, Cairo, Egypt.

#### Light microscopy (LM) (Semithin sections)

Nymphs were dissected out under cold phosphate buffer adjusted to the pH 7.3^50^ and integuments were fixed for 2 h. in 3% cold fresh glutaraldehyde in Invertebrate Laboratory, Zoology Department, Faculty of Science, Ain Shams University, Cairo, Egypt. They were washed for 30 min. in phosphate buffer, postfixed in cold 1% osmic acid for 2 h. and washed again in fresh buffer for another 30 min. Then they were dehydrated in a graded series of ethanol and kept in pure acetone for 30 min. For infiltration with the resin Epon 812, integument were transferred through mixtures of acetone-Epon 812 in the ratio of 1:1 for 1 h., 1:2 for another 1 h. and then finally embedded in pure Epon 812 mixture in plastic molds kept in an oven at 60 ˚C until polymerized after 24 h. Semithin section (1 μm) from blocks were cut with glass knives using RMC ultratome and stained with 1% toluidine blue stain (TB)^51^ for light microscope examination. Section preparations were done in the Electron Microscope Unit, Faculty of Science, Ain Shams University, Cairo, Egypt.

Although this study didn’t have neither experimental higher invertebrate or vertebrate animals nor humans, it was ethically approved by the Research Ethics Committee of Faculty of Science, Ain Shams University, Cairo, Egypt, Code: ASU-SCI/ZOOL/2024/11/3. The committee confirmed that all experiments were performed in accordance with relevant guidelines and regulations.

## Results

### Tick identification

**Fig. 1:**
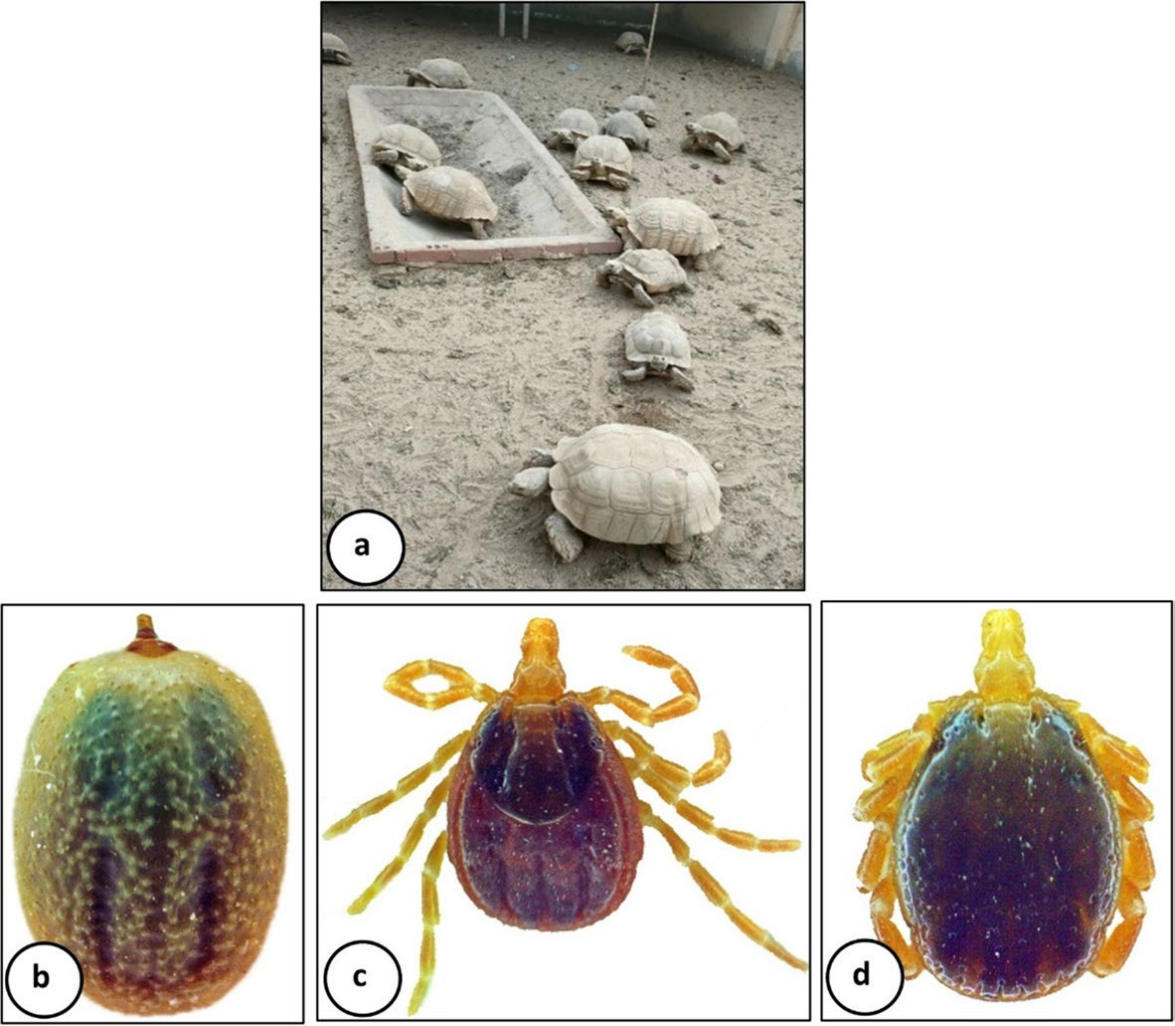
Photomicrograph of *Hyalomma aegyptium*. **a** *Centrochelys sulcate* host. **b** Engorged nymph. **c** Newly hatched female. **d** Newly hatched male.

All tick specimens collected from *Centrochelys sulcate* (Fig. 1a) were identified as *Hyalomma aegyptium* engorged nymphs (Fig. 1b). This identification was confirmed after examination of newly hatched adult females and males (Fig. 1c and d).

### Biological studies

**Fig. 2:**
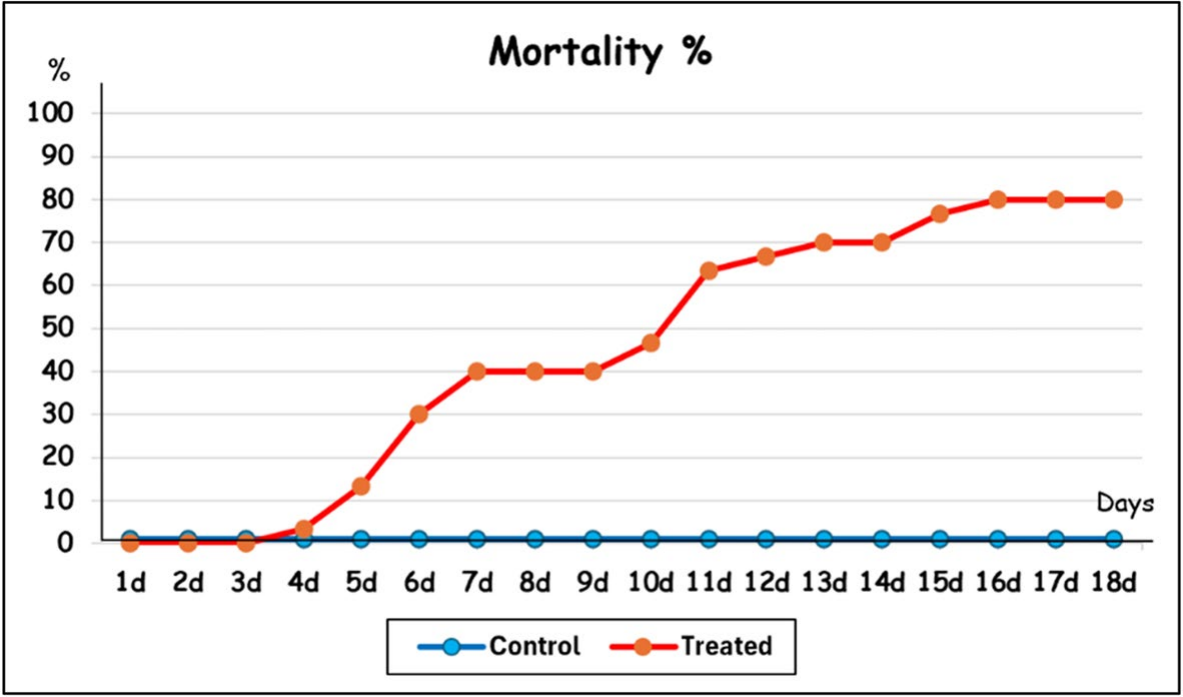
Mortality percentage of *Hyalomma aegyptium* nymphs after treatment with *Chlorella vulgaris* alga for 18 days (d).

The mobile percentage of control nymphs was recorded as 100% from 1st to 18th days, while it decreased from 100 to 20% in treated ones. On the other hand, mortality percentage increased in treated ones being 0–80%, versus 0% in control during the examined period (Fig. 2). It is noticed that the effect of *Chlorella* on nymphal ticks began 4 days after treatment (Fig. 2). Dead nymphs seemed to be extremely hard, concavely curved, with deep dark in color. In addition, it can’t move after forcibly stretched.

The molting period was delayed in treated groups (14.67 d) and showed a highly significant increase (*p* < 0.01) versus control ones (12.7 d) (Table 1). The molting percentage reached 100% in control nymphs compared with only 20% in treated ones after 18 days of treatment (Table 1).

**Table 1:** Molting period and percentage of *Hyalomma aegyptium* nymphs after treatment with *Chlorella vulgaris* Alga for 18 days.

	Molting period (Days) Mean ± SE (Min-Max)	Molting %									
		9d	10d	11d	12d	13d	14d	15d	16d	17d	18d
Control	12.7 ± 0.29 (9-14)	13.33	13.33	13.33	23.33	66.67	100	100	100	100	100
Treated	14.67 ± 0.67 ** (11–15)	0	0	16.67	16.67	16.67	16.67	20	20	20	20

**; Highly significant (*p*<0.01).

SE; Standard error, Min; minimum value, Max; Maximum value, d; days.

The sex ratio of males: females recorded 6:1 after molting of engorged nymphs for control group, while all treated nymphs that succeeded in molting gave molted to males only.

Efficacy of the alga was recorded 44.43% during 18 days after treatment, while it reached 80% at the end of the examined period.

### Histological studies

All treated live nymphs examined in this section suffered from the same algal effects.

#### Morphological studies

**Fig. 3:**
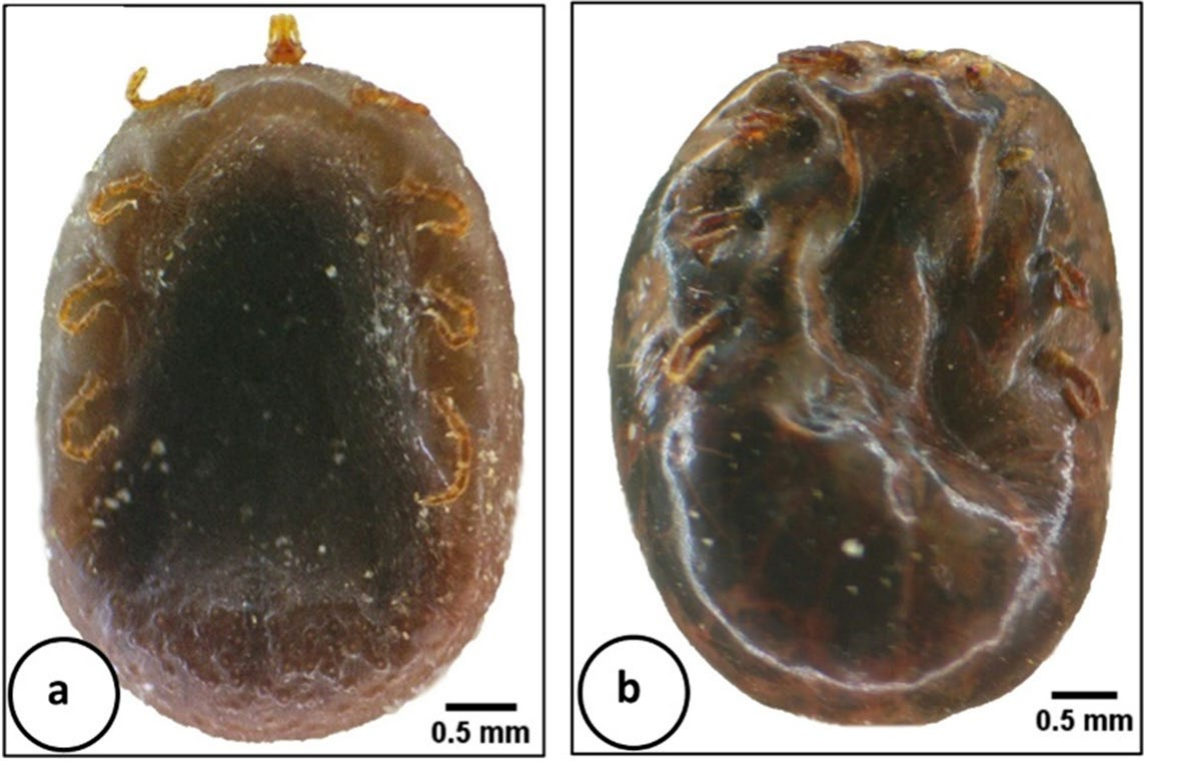
Photomicrograph of *Hyalomma aegyptium* nymph (ventral view) for general observations. **a** Control. **b** Treated with *Chlorella vulgaris*.

Generally, the photomicrographs of *H. aegyptium* nymphs showed that the body of treated one suffered from stiffness compared to control (Fig. 3a and b). The ventral surface of treated nymphs was shrunk with severe corrugation in the upper part of the body (Fig. 3b), while the lower part was smooth compared with the dome-like structures found in the control specimen (Fig. 3a). After treatment, the capitulum shrinkage, and loss of some leg segments were clearly observed (Fig. 3b).

**Fig. 4:**
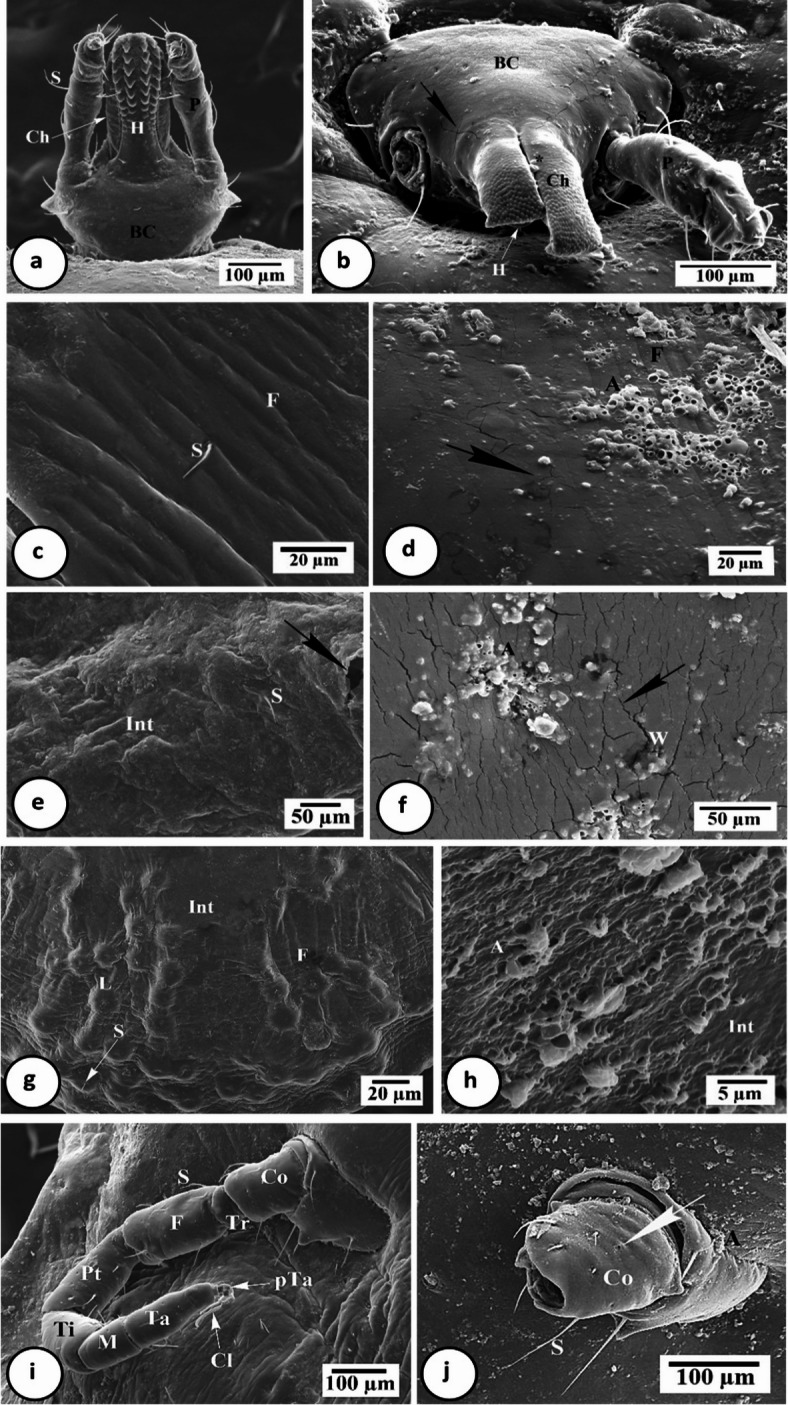
Scanning electron micrographs of *H. aegyptium* nymph body parts. **a** Capitulum of control nymph showing hypostome (H) with laterally located palps (P), basal capitula (BC) and sensillae (S). **b** Capitulum of treated nymph with cracks (arrow) in basal capitula (BC), erected chelicera (Ch) hiding damaged hypostome (H) and loss of one palp (P). **c** Enlarged portion of control nymph integument showing integumental folds (F) with sensillae (S). **d** Enlarged portion of treated nymph integument showing scattered alga powder (A) forming cracks (arrow) with less numerous folds (F). **e** Enlarged portion of nymph integument after 11 days of feeding appeared the beginning of molting process (arrow) with shed of integument (Int). **f** Enlarged portion of nymph integument after 11 days of treatment with embedded alga powder (A) forming warts (W) and increasing cracks (arrow). **g** Enlarged posterior portion of control nymph integument (Int) with folds (F) and lamellae (L) forming dome-like structures bearing setae (S). **h** Enlarged portion of treated nymph integument (Int) with slightly shed parts and molted alga powder (A). **I** Leg of control nymph contains 7 segments; coxa (Co), trochanter (Tr), femur (F), patella (Pa), tibia (Ti), metatarsus (M) and tarsus (Ta), and ended with pretarsus (pTa) and 2 claws (Cl). **J** Leg of treated nymph with scattered alga powder (A), loss of segments except coxal one (Co) that loss some sensillae (S).

Scanning electron microscopical examination showed that the capitulum of treated nymphs was highly deformed and dilated compared to control (Fig. 4a and b). It was invaginated inside the anterior body part (Fig. 4b). Basis capitula suffered from cracks with a broken sensilla. Incomplete development of erected chelicerae was observed, with hiding the hypostomes that seemed to be damaged (Fig. 4b). Palp appeared dilated, erected, fragile, and a single of its pair was lost.

The alga powder became incorporated into the integumental surface leading to numerous crakes with less number of foldings, giving it a dry, cracked landscape appearance with wart-like projections (Fig. 4d and f), versus observed in the control (Fig. 4c). After ⁓11 days of feeding, nymphs began to molt as an old integument appeared as sheath and shed from underlying new one (Fig. 4e). In addition, the integument showed lamellae forming dome-like structures bearing setae at the posterior body part (Fig. 4g). On the other hand, the integument of treated nymph began to shed intermittently in certain areas, forming a fragile, feathery layer in the presence of molten alga powder (Fig. 4h).

The legs of treated nymphs appeared fragile as they were lost, especially from 2nd trochanter segment (Fig. 4J) in contrast to control that had 7 segments ending in 2 claws (Fig. 4I). The alga powder was found embedded within the articulated parts of the legs (Fig. 4J). Additionally, the coxal segment showed a noticeable reduction in some sensilla.

#### Light microscopical studies (Semithin sections)

**Fig. 5:**
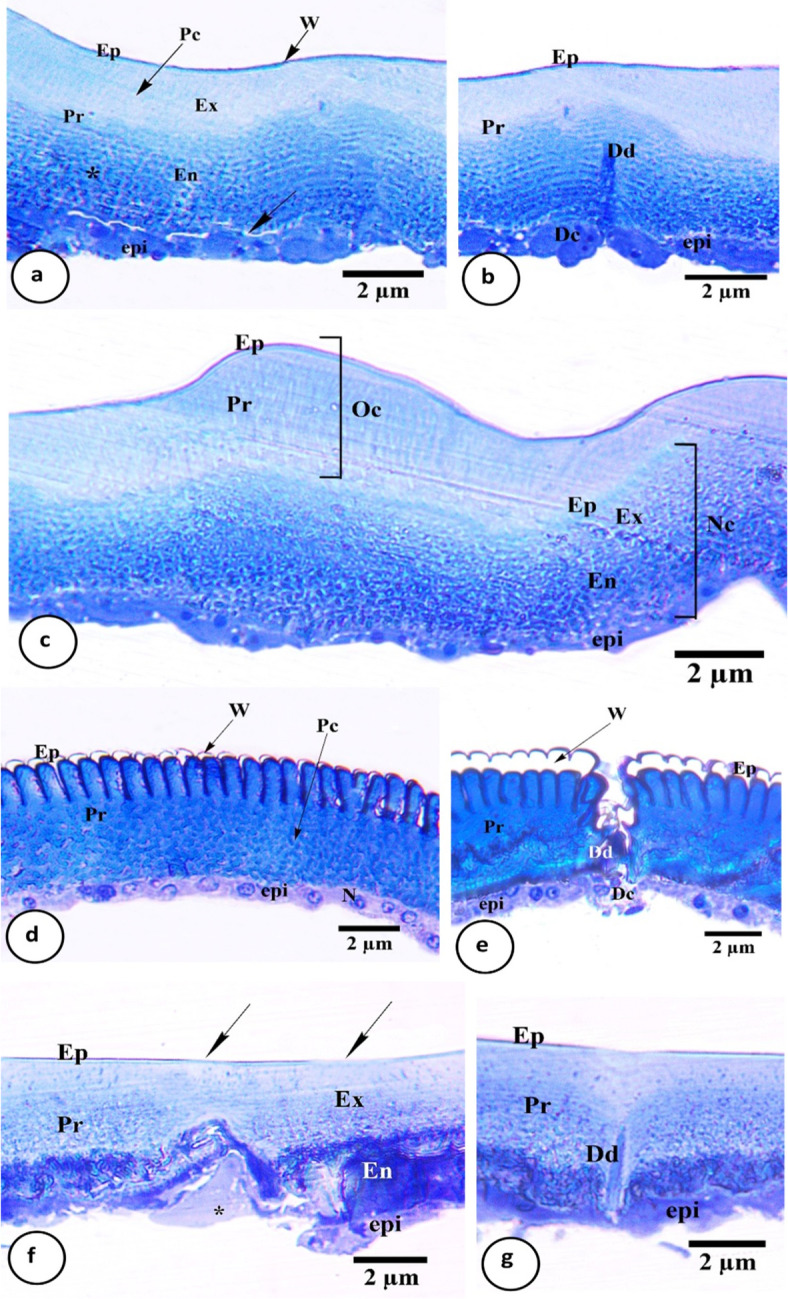
Semithin sections of the integument of *Hyalomma dromedarii* nymph. **a** Integument before molting, showing epicuticle (Ep) covered with wax layer (W), procuticle (Pr) that consists of exocuticle (Ex) with pore canals (Pc) and endocuticle (En) with lamellar organization (asterisk), epidermis (epi) and exuvial space (arrow). **b** Dermal gland before molting consists of dermal duct (Dd) and 2–5 dermal cells (Dc), epicuticle (Ep), procuticle (Pr) and epidermis (epi). **c** integument during molting with formation of new cuticle (Nc) with epicuticle (Ep), exocuticle (Ex), endocuticle (En) and epidermis (epi), and shedding of old one (Oc) with epicuticle (Ep) and procuticle (Pr). **d** Integument after molting, epicuticle (Ep) covered with thick wax layer (W), undifferentiated procuticle (Pr) with pore canals (Pc), and single epidermal layer (epi) with large nucleus (N). **e** Dermal gland after molting consists of wide dermal duct (Dd) and 2–5 small dermal cells (Dc), epicuticle (Ep) with thick wax layer (W), procuticle (Pr) and epidermis (epi). **f** Treated integument with eroded areas (arrows) in epicuticle (Ep), procuticle (Pr) with disorganized exocuticle (Ex) and coagulated endocuticle (En), and damaged epidermis (epi) that lysed in some areas (asterisk). **g** Treated dermal gland with erected duct (Dd), epicuticle (Ep), procuticle (Pr) and epidermis (epi).

The integument of nymph *H. aegyptium* consists of a cuticular layer underlies with epidermis (Fig. 5a). The cuticle is divided into an outer thin epicuticle, and an inner thick lamellated procuticle. The latter one presents two well defined regions; the exocuticle (next to the epicuticle) and the endocuticle (adjacent to the epidermis). Pore canals in the procuticle are continuous with short wax canals traverse the epicuticle. Epidermis is a single layer of cuboidal to oval cells that secretes the cuticle. The nucleus occupies most of the cell with strongly stained affinity (Fig. 5a-e).

After blood feeding, the cuticle stretches, that epicuticle appeared straight with minor folds, and integument became ready for molting process preparation (Fig. 1a and b). At the beginning of this process, the epidermal cells increased in volume, became flat and started to divide (Fig. 5a). The additional procuticle lamellae were deposited, and pore canals lose their parallel pattern. Then the epidermis detached from the cuticle (apolysis) forming exuvial space that became filled with molting fluid (Fig. 5a). Dermal glands occur throughout the epidermal layer (Fig. 5b). Each gland consists of 2–5 large polygonal cells that are characterized by their irregular shape and connected to the exterior via a fine duct. The dermal duct filled secretion during this stage (Fig. 5b). After that, epidermis deposited the second epicuticle layer with many wax filaments. The epidermis deposited lamellated procuticle with pore canals connected to wax filaments of epicuticle. Staining affinity of endocuticle was stronger than exocuticle layer (Fig. 5c). At this stage, the digestion of old cuticle began (disorganization of inner most procuticle layer and resist digestion of epicuticle and adjacent procuticle lamellae) (Fig. 5c). This resistance will shed with molting membrane after absorption of molting fluid (ecdysis). Epidermal cells became single layer and flat in shape (Fig. 5c).

After molting, parallel, extensible epicuticular deep folds appear (zigzag model) (Fig. 5d). The outer most layer of epicuticle, wax layer, was thick and highly clarified. The procuticle regions were less differentiated (Fig. 5d). The pore canals filled with secretion. The epidermal cell volume became cuboidal, back to its normal size and weekly stained with clearly oval nucleus. Dermal gland seemed to be dilated with empty duct, and decreased cell volume (Fig. 5e).

Generally, *C*. *vulgaris* caused cuticle disorganization, and damages in the epithelial cells (Fig. 5f). Cuticle thickness decreased compared to control. Although cuticle subdivisions were distinguished, each layer was highly destructed. The epicuticle was rarely folded and suffered from erosion in some areas (Fig. 5f). The procuticle presented a single, little thick compact layer, without lamellar organization. Damaged pore canals in exocuticle that lost their organization were found. Endocuticle suffered from coagulation or coalesced as it appeared compact solid layer without details (Fig. 5f). Epithelium was interrupted in some areas (Fig. 5f). Damaged and/or dilated epithelial cells with fragmented nuclei were observed, and some were lysed (Fig. 5f and g). It is worth noting that neither formation of new cuticle nor ecdysis process occurred. Dermal duct appeared as solid erected stalk filled with coagulated secretion (Fig. 5g). Dermal cells were completely lysed or degenerated.

## Discussion

### Tick identification

In agreement with the current study’s observations, only *H. aegyptium* nymphs have been recorded and collected from *Testudo graeca*^52–55^ and *T. hermanni*^23^ in Turkey. On the other hand, Hoogstraal and Kaiser^14^, Hoogstraal et al.^56,57^, Sweatman^58^ and Walker et al.^59^ reported their collection from small mammalian and bird species.

Hoogstraal^44^ and Hoogstraal and Kaiser^60^ stated that *H. aegyptium* was introduced into Egypt but did not become established in it. The presence of ticks on *T. graeca* and *T. marginata* in Poland originating from Egypt was not confirmed^61^. However, in 1989^62^ Liebish and colleagues updated Egypt’s tick checklist, recognizing *H. aegyptium* as an endemic species. Additionally, Clark and Doten^63^ reported its finding on tortoises in Florida imported from Egypt, raising its possible occurrence in Egypt^64^. Since then, surveillance of *H. aegyptium* in animals imported to Egypt has been limited. In this study, *H. aegyptium* engorged nymphs were found on the African spurred tortoise, emphasizing the importance of monitoring this tick species within Egypt.

Hoogstraal et al.^57^ found that immature stages of *H. aegyptium* enter Egypt through bird migration and may occur on quail, pigeon, chats, and warblers^65^. Nowak-Chmura^66^ suggested that the tortoises with the ticks feeding on them, were brought to Poland by tourists or during the inspection of a consignment of tortoises, intended for sale and terrarium-breeding. In Italy, illegally brought tortoises from North Africa were contaminated with *H. aegyptium* males, females, and nymphs^67^. The previous possibilities may be the same that facilitating the entrance of this tick species to Egypt.

In recent decades, biological control strategies for tick management have significantly improved, especially due to the increasing resistance to chemical acaricides, and providing sustainable, environmentally friendly methods^68–70^. Previous studies have demonstrated that biological control agents exhibit potent acaricidal activity against various species of Ixodid ticks^71^. For example, entomopathogenic fungi (*Beauveria bassiana* and *Metarhizium anisopliae*)^72–78^and plant extracts^71,79–84^ served as effective tick control alternatives. However, microalgae have a potential effect against several mosquito vectors^85–87^ the use of microalgae as a biological control agent against ticks is limited. Therefore, this study aims to assess the acaricidal effectiveness of *Chlorella vulgaris* algae powder against the engorged nymphal stage of *H. aegyptium*.

### Biological studies

From the present work, *C. vulgaris* effects began 4 days after treatment of *H. aegyptium* nymphs. It caused a high mortality percentage reached 80%, with 80% efficacy after examined period.

The data available for the control of *H. aegyptium* was very limited. The only two studies reported by Laghzaoui et al.^26^ and El-Mustapha et al.^88^ showed that essential oils derived from different plant species were toxic to nymphs with high mortality. They revealed that the nymphal stages exhibited the highest susceptibility to essential oils, compared to the other immature stages. Moreover, the present work suggested that as structures are changing very rapidly after feeding nymphs, this stage could be easily affected.

Molting period was delayed with a 20% molting percentage after 18 days of treatment. All treated engorged nymphs that succeeded molting, resulted in males only versus control that molted into number of males more than females. Similar to the present results, the only data was reported by Kalmár et al.^22^. They found that from 8 fully engorged *H. aegyptium* nymphs, 6 males and 2 females resulted after molting.

Several studies determined the sex ratio of adult male and female *H. aegyptium* directly collected from their hosts^17,22,24,55,67,89–94^. They found that tortoises were significantly more infested by male ticks than females. This could be explained by the fact that male ticks spend more time than females on their hosts and mate with one or several females^90,95^. In addition, Ali and Taha^83^ concluded that males were more resistant than females, as females were exhausted during the egg production process^96^ with a decreased immunity, which may be explained increasing male number.

### Histological studies

The present histological studies were represented 11 days after feeding and/or treatment of nymphs. According to biological results, treated nymphs began to molt after 11 days of treatment. So, it is easy to compare between treated and untreated nymphs.

From photomicrographs in the present work, *H. aegyptium* treated nymphs were dark in color with rigid and corrugated integument. The capitulum was deformed, and legs lost some segments.

This study is the first to include high-quality photomicrographic images of *H. aegyptium* nymphs to aid in their identification. On the other hand, the previous ones published photomicrographs of *H. aegyptium*, especially males and females, for general observation and classification without any description^20,55,66,94,97–103^.

The current results using SEM showed that capitulum, integument and legs of treated engorged nymphs suffered from extreme damage.

Invaginated capitulum was clearly observed in treated nymphs. This phenomenon may be considered as a defense mechanism (a physiological response to the stress caused by the alga treatment) resulting in decreasing the surface area facing alga treatment. Protecting mouth parts and reducing the possibility of alga entry are the other suggested reasons for this retraction. Abnormalities have been observed in the chelicera and hypostome regions. This may indicate that, despite their defensive efforts, the treatment may still inflict significant damage, affecting critical feeding structures necessary for their survival.

The treated integument had a dry, cracked landscape appearance with wart-like projections. It sheds intermittently in certain areas, forming a feathery layer in the presence of molten alga powder. It was suggested that *Chlorella* powder may affect the epicuticle layer of the integument succeeding in embedding inside forming warts. The molten alga powder may be the result of absorbing moisture or some integumental contents, especially lipids^104^ which lose their protective and water balance function^105,106^ leaving it dry with cracks formation.

The legs of treated nymphs appeared fragile as they were lost, especially from 2nd trochanter segment. As a very limited movement segment and attached to insect’s body^107^ Coxa is the strongest segment that wasn’t easily lost versus the other movable ones.

Experimental amputation of palps in mouthparts and legs of fully engorged nymph *H. aegyptium* resulted in regeneration of normal ones, but legs were shorter and subnormal sized after molting^108^. So, it may be suggested that all lost parts from treated nymphs in the present work couldn’t be recovered as there was neither molt nor immunity found. Even if this happens, the resulting abnormalities will prevent normal live and life cycle.

Integument acts as an exoskeleton that covers the tick body and minimizes the rate of water loss to prevent desiccation^109,110^. It provides protection against external agents such as predators, mechanical shocks, and climatic adversity^111,112^ however it does not allow the growth of the individuals^109^.

From the present study, cuticle disorganization, and epidermal destruction were observed after treatment. The data about the histological observation of tick integument after treatment was scarce. Like the present results, Fluazuron (arthropod growth regulator) induced morphological integument changes in *R. sanguineus* nymphs^113^. They reported not only cuticle disorganization but also absence of its subdivisions and damages in the epithelial cells using highest concentrations. The epicuticle and procuticle (exocuticle and endocuticle) are thinner with non-continuous epidermal cells^114^. On the other hand, they reported in 2014b^113^ undamaged epithelial cells after Fluazuron treatment with low concentrations.

From the current results, the cuticle decreased in thickness after treatment. It could be suggested that damaged epidermal cells that are responsible for the formation of cuticular layers, also the damaged disorganized pore canals in exocuticle that transport materials from epidermis to outside could be the reasons. The deficiency in the amount of chitin would prevent the increased growth of the cuticle, fundamental process for the accommodation of the great amount of blood ingested by the tick during engorgement^111,115,116^ as well as the correct deposition and organization of the chitin to ensure the correct deposition^117^. Similar results were found by de Oliveira et al.^113,114^ for *R. sanguineus* and Mommaerts et al.^118^ and Saenz-De-Cabezon et al.^119^ for *Ctenocephalides felis* fleas, who observed decreased thickness and also loss of procuticle subdivisions after the treatment with fluzaron and lufenuron, respectively. Although cuticle subdivisions were distinguished, each layer was highly destructed. This differentiation is backed by the treatment exhibited after feeding as all cuticle layers were found in old cuticles.

The epicuticle was rarely folded, indicating that nymph couldn’t digest blood meal and so couldn’t be molted. Erosion found in some areas of this layer could increase the loss of water, making desiccation for ticks and finally death.

The procuticle without lamellar organization and coalesced endocuticle were clearly investigated. This may be due to interference of *Chlorella* in the synthesis and/or deposition of chitin that leads to abnormal endocuticular deposition which affects the cuticle’s elasticity and stiffness, preventing the normal formation of a new cuticle and the completion of ecdysis^120–123^.

Damaged epithelial cells were clearly observed after treatment in the present results. Similar results were reported by de Oliveira et al.^114^. de Oliveira et al.^113^ suggested that this destruction would prevent the secretion of elements to synthesize new cuticle, leading to less resistant individuals and less able to survive.

Dermal gland suffered from solid erected duct filled with coagulated secretion, with degenerated dermal cells. This may explain that the secretions from these glands may be affected by treatment which indirectly affects the other cuticular parts. Another explanation is that it would be a defense mechanism as Balashov^124^ and Walker et al.^125^ suggested this secretory material could block the ducts against loss of water vapor.

Due to all previous integumental destruction, neither formation of new cuticle nor ecdysis process occurred. Similar results reported by other authors^109,113,114,126,127^. This may indicate that *C. vulgaris* is inhibiting the processes related to the molting of nymphs probably by preventing the synthesis and/or deposition of the necessary chitin to form a new cuticle that will cover their body during periodic ecdysis. According to Palli and Retnakaran^122^ this prevention would occur by the inhibition of certain biochemical processes, such as chitin synthase, proteases that activate chitin synthetase and the activation of chitinases involved in the catabolism of chitin. de Oliveira et al.^113^ reported that this damage was so serious that even if ticks can complete the ecdysis process and advance to the next stage they will not be able to survive, once their cuticle is very vulnerable and thus unable to protect them against predators and to reduce the water body loss^111,116^.

Additionally, the importance of the ecdysteroids in the hormonal control of the molting process in arthropods is well documented^128,129^. In ticks, exogenous ecdysteroids are known to exert physiological effects including molting^130^. From the previous data, it was suggested that *C. vulgaris* may affect the central nervous system of nymphal ticks that affect hormonal secretions (either deficiency or differences in the time needed to express the response to the hormonal stimulus), which indirectly leads to inhibition of ecdysis. Germond et al.^130^ investigated that blood meal induces the processes which lead to the deposition of the new cuticle and to ecdysis. This could be another explanation that *Chlorella* may enter mouth parts and affect the gut, and digestive process, and indirectly affect the ecdysis.

Although Laghzaoui et al.^26^ and El-Mustapha et al.^88^ were the only available data evaluating acaricidal properties of essential oils from Moroccan plants against immature ticks of *H. aegyptium*, they ignored their histopathological effects on the stages. To our knowledge, this is the first work studying the histopathological effect of *C. vulgaris* algae as a biological control agent against the engorged *Hyalomma* nymphs, emphasizing histopathological changes in the integument due to the histological technique is valuable for studying tick morphophysiology, aiding in assessing synthetic and natural acaricides’ effects at tissue and cellular levels^131^.

Summarily, the authors suggested that *Chlorella* powder has 4 possible mechanisms to make it harmful to ticks. The first mechanism is that alga powder can enter the tick body through either damaged integument or body openings (mouth parts, spiracles, genitalia, anus), circulating in the hemolymph, and damaged internal organs, especially nervous system through interfering in chitin metabolism or in the production of hormones responsible for molting and/or gut that affect blood digestion involved in ecdysis. Kang et al.^132^ reported that some *Chlorella* strains contain toxins and accumulate heavy metals from their environment^133^ that affect living organism’s health and survival. This could be the second mechanism in the current work.

The third one is the fact that *Chlorella* cells are rich in minerals and proteins and can accumulate high amounts of lipids^104,134^. This supports that any excess of it, in the current study through treatment, resulted in extreme damage. As lipids, combined with protein, serve to stiffen the insect’s cuticle^135^ any increase in their levels resulted in more integumental rigidity with loss of its function. In blood sucking arthropods, Whiten et al.^136^ found that if iron and heme didn’t control, it may cause oxidative damage, protein degradation, and cell death especially in the gut. Also increased minerals in the current study may contribute to neurotoxicity. Mossa et al.^137^ reported that exposure to high doses of insecticide-containing minerals causes neurotoxicity and kills insects by an effect on their nervous system. Additionally, Pradhan et al.^138^ reported that if *C. vulgaris* was taken at limited level, it can stimulate the immunity of the organism. In the present study, the increase of alga powder may decrease the tick immunity as a fourth mechanism, exposing them to infection and easy damage.

All the previous mechanisms, whether individually or combined, lead to morphological abnormalities and morphophysiological changes during the development and metamorphosis that can impede various tick activities, including feeding and movement, and inhibit emergence of new individuals that cut the life cycle and ultimately resulting in death. This indicates the possibility of using this alga in the control of this stage of the biological cycle of *H. aegyptium*.

This would provide critical information that could help in the development of new methods to control *H. aegyptium* ticks and/or improve the existing methods of control, making them more specific, less toxic, and less harmful to the environment and non-target organisms and less resistant inducing for the ticks.

## Conclusions and future work

Limiting illegal trade or importing animals before examining them or using safer materials for the exotic tick species must be paid attention. This study is the first to demonstrate the biological and histopathological effects in *Hyalomma* nymphs after treatment with *Chlorella*. The effect of *Chlorella* was either mechanically through powder particles or physiologically through effect on organs, preventing the emergence of adults and finally led to death. The study of tick histology has become a critical tool to understanding the action of used alga and generate information that can help researchers to better understand the biology of these ectoparasites. More studies on this matter are needed for a better understanding of the role of microalgae as biological agents in controlling ticks. Further histological and molecular works required to study the effects of this alga on the newly molted adults, either males or females after nymphal treatment. Different biotic conditions will be useful for understanding the effect of *Chlorella* on *Hyalomma* ticks at field levels. This study may provide important information as a basis for future studies which need to understand the main organ systems of these ectoparasites to develop more specific and efficient methodologies of tick control.

## Data Availability

We are the authors assure that all data and materials support the published claims and comply with field standards. The data are mentioned in the manuscript and will be available after publication.
